# Outcomes of the resuscitative and emergency thoracotomy at a Dutch level-one trauma center: are there predictive factors for survival?

**DOI:** 10.1007/s00068-022-02021-x

**Published:** 2022-06-17

**Authors:** A. S. Y. Sam, F. Nawijn, K. E. M. Benders, R. M. Houwert, L. P. H. Leenen, F. Hietbrink

**Affiliations:** Department of Surgery, University Medical Center Utrecht, Utrecht, the Netherlands

**Keywords:** Emergency thoracotomy, Resuscitative thoracotomy, Trauma, Thoracic trauma

## Abstract

**Purpose:**

To investigate the 30-day survival rate of resuscitative and emergency thoracotomies in trauma patients. Moreover, factors that positively influence 30-day survival rates were investigated.

**Methods:**

A retrospective study of patients (> 16 years), between 2008 and 2020, who underwent a resuscitative or emergency thoracotomy at a level-one trauma center in the Netherlands was conducted.

**Results:**

Fifty-six patients underwent a resuscitative (*n* = 45, 80%) or emergency (*n* = 11, 20%) thoracotomy. The overall 30-day survival rate was 32% (*n* = 18), which was 23% after blunt trauma and 72% after penetrating trauma, and which was 18% for the resuscitative thoracotomy and 91% for the emergency thoracotomy. The patients who survived had full neurologic recovery. Factors associated with survival were penetrating trauma (*p* < 0.001), (any) sign of life (SOL) upon presentation to the hospital (*p* = 0.005), Glasgow Coma Scale (GCS) of 15 (*p* < 0.001) and a thoracotomy in the operating room (OR) (*p* = 0.018). Every resuscitative thoracotomy after blunt trauma and pulseless electrical activity (PEA) or asystole in the pre-hospital phase was futile (0 survivors out of 11 patients), of those patients seven (64%) had concomitant severe neuro-trauma.

**Conclusion:**

This study found a 30-day survival rate of 32% for resuscitative and emergency thoracotomies, all with good neurological recovery. Factors associated with survival were related to the trauma mechanism, the thoracotomy indication and response to resuscitation prior to thoracotomy (for instance, if resuscitation enables enough time for safe transport to the operating room, survival chances increase). Resuscitative thoracotomies after blunt trauma in combination with loss of SOL before arrival at the emergency room were in all cases futile, interestingly in nearly all cases due to concomitant neuro-trauma.

**Supplementary Information:**

The online version contains supplementary material available at 10.1007/s00068-022-02021-x.

## Introduction

A resuscitative or emergency thoracotomy can be a salvage procedure to resuscitate severely injured patients presenting in extremis (i.e., resuscitative thoracotomy in case of pulseless electric activity (PEA), asystole, severe (hemorrhagic) refractory shock or pericardial tamponade) or to treat life-threatening injuries, such as a massive hemothorax or removal of an object penetrating the thorax (i.e., emergency thoracotomy if patient is hemodynamically stable at presentation or fluid responsive). Both are part of the damage control surgery arsenal and are usually performed in the emergency department (ED) or the operating room (OR) [1–3]. Furthermore, especially resuscitative thoracotomies are increasingly applied in the field by Helicopter Emergency Medical Services (HEMS) [4–6]. While the objective of a damage control thoracotomy is undisputed, the indications remain a topic of debate and are closely related to the chance of survival.

Consensus exists that resuscitative and emergency thoracotomies have higher survival rates after penetrating injury when compared to blunt injury [3, 7–9]. However, these survival rates still differ notably between studies and are greatly influenced by the indication for the thoracotomy, which is subject to the country in which it is performed [8, 10]. For example, a large trauma registry from the United States including resuscitative thoracotomies performed in the emergency department report a survival rate of 3% for blunt trauma and 14% for penetrating trauma [10]. In comparison, a systematic review on resuscitative and emergency thoracotomies performed in both the ED and OR from centers across Europe reported survival rates are as high as 25% in blunt trauma and 62% in penetrating trauma [8].

**Fig. 1:**
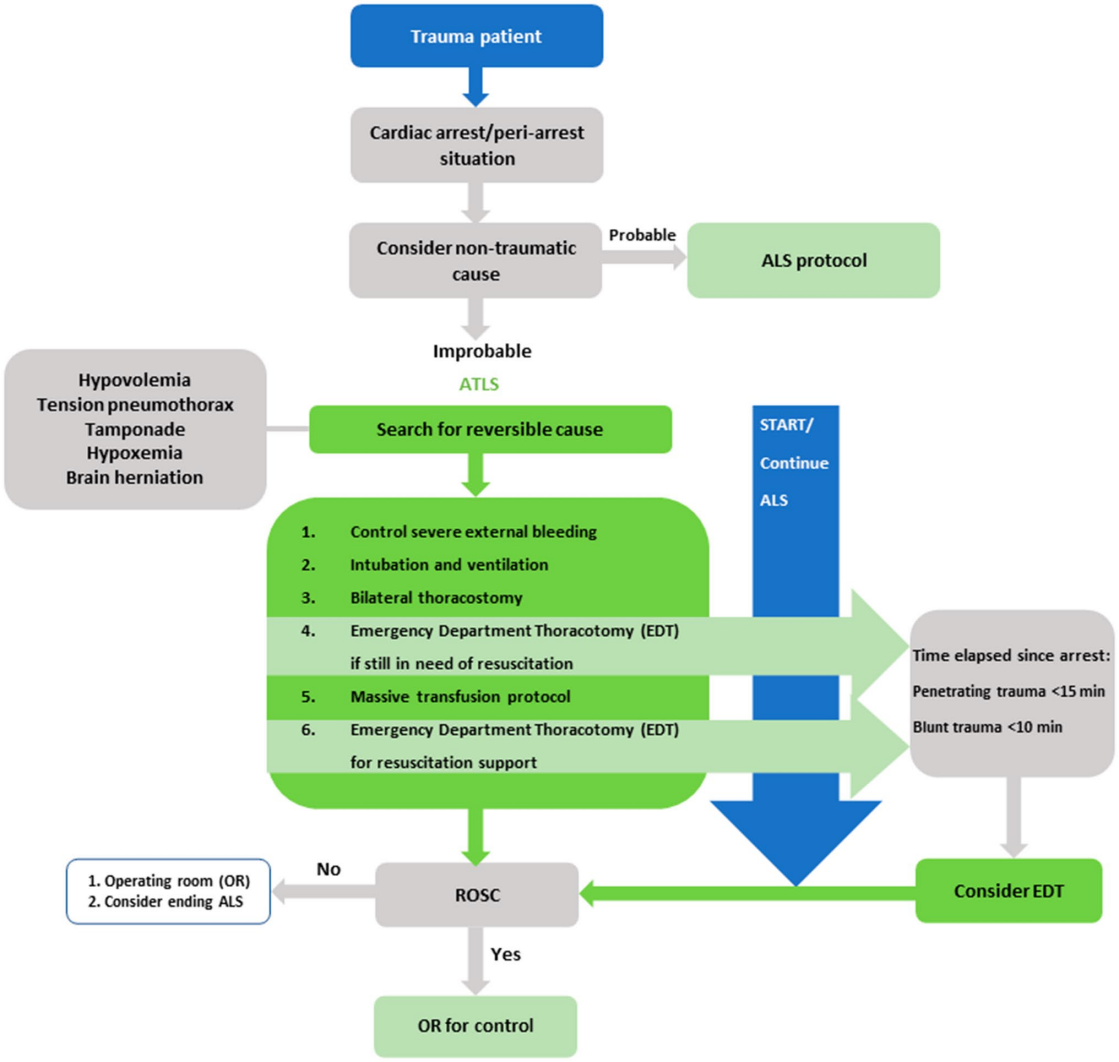
Flowchart of decision-making process for trauma patients with cardiac arrest in the emergency department at study hospital

Consequently, the Western Trauma Association formulated within their guidelines that a thoracotomy for blunt trauma is only indicated for patients arriving at a trauma center with signs of life (SOL), and/or have a witnessed cardiac arrest < 10 min prior to arrival. For patients with penetrating thoracic injuries, the indication is stretched to cardiac arrest < 15 min prior to arrival and witnessed arrest [11, 12]. As a result of the importance of elapsed time after cardiac arrest and the trauma mechanism, the decision to perform a resuscitative or emergency thoracotomy will highly depend on geographic circumstances and pre-hospital logistics [6, 13, 14]. For example, within the Netherlands, resuscitative or emergency thoracotomies by the HEMS are rare since the Netherlands has 11 level I trauma centers, enabling relatively short transport times to level I trauma centers[5]. In accordance with established recommendations, our trauma center developed a flowchart for guiding the decision-making process in the ED for injured patients presenting in extremis, specified for the local situation (Fig. 1) [15]. Hence, this study investigated the outcomes of resuscitative and emergency thoracotomies in trauma patients at our Dutch level 1 trauma center in relation to the 30-day survival. Moreover, factors that positively influence 30-day survival rates were investigated.

## Methods

The institutional review board of the medical ethics committee at our hospital provided a waiver for retrospective data collection. This retrospective cohort study was reported according to the Strengthening the Reporting of Observational Studies in Epidemiology (STROBE) guideline [16]. A study protocol was written and stored at the study hospital.

### Study design

Trauma patients aged 16 years and older, who underwent a resuscitative or emergency thoracotomy between January 2008 and December 2020 at our level 1 trauma center were included in this study. The difference between resuscitative and emergency thoracotomy was based upon the indication: resuscitative thoracotomies were defined as performed in patients in extremis, due to pulseless electric activity (PEA), asystole, severe (hemorrhagic) refractory shock or pericardial tamponade. Emergency thoracotomies were performed in patients deemed as hemodynamic (transient) responder in case of massive hemothorax or retrieval of foreign (penetrating) object from the thorax. All patients that were operated on in the OR, were first seen in the ED, there is no OR-first protocol in our center. Within our center, all thoracotomies were performed by trauma surgeons. In the Netherlands, trauma surgeons are trained in treating both visceral trauma as well as orthopedic fractures. Patients were identified retrospectively within the hospital administration database using the surgical procedural codes for thoracotomies. Electronic patient charts were assessed for eligibility, after which all parameters and outcomes were extracted and collected. The study size was determined by the number of eligible patients within the study period.

### Data collection

Patient characteristics (age, sex), variables regarding the pre-hospital condition (SOL, PEA, asystole), vital signs upon arrival, blood results, trauma characteristics (injuries, mechanism of injury, injury severity score (ISS)) and treatment characteristics (indication thoracotomy, approach of thoracotomy and location where thoracotomy was performed, concomitant laparotomy) were collected. SOL was defined as presence of reactive pupils, spontaneous movement, spontaneous respiration, presence of carotid pulse, or measurable blood pressure.

The primary outcome was 30-day survival. For survivors, admittance and length of stay in the intensive care unit (ICU), the total length of hospital stay (LOS), complications and subsequent operations were collected. Neurological outcome of survivors was assessed using the Glasgow Outcome Scale, this scale uses scores ranging from 1 to 5 with 1 being death and 5 being mild or no neurological disability [17].

For non-survivors, the location of death (ED, ICU or OR), cause of death and the possibility of survivability (as discussed in a mortality and morbidity meeting) were also collected. All non-survivors were categorized in one of the following groups:

- Non-survivable: injuries not compatible with life (i.e., aortic root rupture, complete coronary arteries transection).
- Beyond survival: patients with injuries that might be survivable as an individual injury, however, non-survivable at time of presentation due to their combination with multiple bleeding sources in chest/abdomen/pelvis, and accompanying factors, such as time since loss of SOL and lethal triad.
- (Active) cessation of treatment after family consultation in combination with pre-injury comorbidities and condition.
- Possible preventable deaths: patient with potentially survivable injuries who could potentially have survived (with the right decisions and/or procedures at the right time after presentation).
- Not trauma-related death: patients who presented as trauma patients but did not die as result of traumatic injuries.

### Statistical analysis

Parametric continuous variables are presented with means and standard deviations (SD), nonparametric continuous variables as medians with interquartile ranges (IQR) and categorical variables as frequencies with percentages. Dichotomous independent variables were analyzed using Chi-square and Fisher’s exact tests, nominal independent variables using the Kruskal–Wallis test and continuous independent variables using the Student’s *t* test or Mann–Whitney U test (based on normality). A *p *value < 0.05 was considered statistically significant. Missing data were handled using pairwise deletion in the statistical analysis. SPSS was used for statistical analysis (IBM Corp. Released 2017. IBM SPSS Statistics for Windows, Version 25.0. Armonk, NY: IBM Corp.).

## Results

A total of 56 patients underwent either a resuscitative or emergency thoracotomy and were eligible for inclusion. The patients were predominantly male (*n* = 46, 82%) and had a median age of 35 years (IQR 23–68). Blunt trauma was the most common trauma mechanism (*n* = 38, 68%) (Table 1). Of the patients with stab wounds, five patients (29%) had multiple stab wounds to the chest and four patients (23%) had a stab wound to the abdomen. All patients with stab wounds to the abdomen required a resuscitative thoracotomy of which two patients survived. The only patient with gunshot wound had multiple gunshot wounds to the chest and abdomen.

**Table 1:** Baseline characteristics of patients undergoing resuscitative or emergency thoracotomy

		Total n = 56 (100%)		Survived first 30 days *n* = 18 (32%)		Deceased within first 30-days *n* = 38 (68%)	*p-*value	Resuscitative thoracotomy *n* = 45 (80%)			Emergency thoracotomy *n* = 11 (20%)	*p-*value	
Age in years, median (IQR)		35 (23–68)		36 (22–61)		34 (23–70)	0.902	34 (22–66)			51 (23–74)	0.212	
Sex, *n* (%)													
Male	46 (82)		17 (37)		29 (63)		0.143		35 (76)	11 (24)			0.183
Female	10 (18)		1 (10)		9 (90)				10 (100)	0 (0)			
ISS, median (IQR)	26 (21–36)		25 (16–30)		29 (24–38)		0.112		29 (22–36)	25 (16–29)			0.139
Trauma mechanism, *n* (%)													
Blunt trauma, n (%)	38 (68)		5 (23)		33 (87)		** < 0.001**		34 (89)	4 (11)			**0.027**
* Motor vehicle accident*	18 (47)		*4 (22)*		*14 (78)*				*16 (89)*	*2 (11)*			
* Bike accident*	6 (16)		*0 (0)*		*6 (100)*				*6 (100)*	*0 (0)*			
* Pedestrian accident*	3 (8)		*0 (0)*		*3 (100)*				*3 (100)*	*0 (0)*			
* Fall from height*	6 (16)		*0 (0)*		*6 (100)*				*6 (100)*	*0 (0)*			
Penetrating trauma, n (%)	18 (32)		13 (72)		5 (18)				11 (61)	7 (39)			
* Stab*	17 (94)		*13 (76)*		*4 (24)*				*10 (59)*	*7 (41)*			
* Gunshot*	1 (6)		*0 (0)*		*1 (100)*				*1 (100)*	*0 (0)*			
Condition upon arrival hospital, *n* (%)													
SOL	39 (70)		17 (44)		22 (56)		**0.005**		28 (72)	11 (28)			**0.024**
* If SOL, heart rate in beats/minute, median (IQR)*^*b*^	*90* ± *39*		*96* ± *33*		*86* ± *44*		*0.363*		*85* ± *43*	*102* ± *24*			*0.273*
* If SOL, heart rate in beats/minute, median (IQR)*^*b*^	*110 (90–130)*		*110 (94–134)*		*107 (85–130)*		*0.676*		*115 (90–135)*	*104 (94–110)*			*0.242*
PEA	8 (14)		1 (13)		7 (87)				8 (100)	(0)			
Asystole	9 (16)		0 (0)		9 (100)				9 (100)	(0)			
GCS, median (IQR)^c^	3 (3–14)		15 (11–15)		3 (3–3)		** < 0.001**		3 (3–8)	15 (15–15)			** < 0.001**
Indication thoracotomy, *n* (%)													
Resuscitative thoracotomy	45 (80)		8 (18)		37 (82)		** < 0.001**		45 (100)	0 (0)			NA
* PEA/asystole*	*23 (51)*		*1 (4)*		*22 (96)*				23 (51)	–			
* Refractory (hemorrhagic) shock*	*19 (42)*		*5 (26)*		*14 (74)*				19 (42)	–			
* Pericardial tamponade*	*3 (7)*		*2 (67)*		*1 (33)*				3 7)	–			
Emergency thoracotomy	11 (20)		10 (91)		1 (9)				0 (0)	11 (100)			
* Massive hemothorax*	*9 (75)*		*8 (89)*		*1 (11)*				–	9 (82)			
* Foreign object in thorax*	*1 (8)*		*1 (100)*		*0 (100)*				–	1 (9)			
* Pericardial injury without tamponade*	*1 (8)*		*1 (100)*		*0 (100)*				–	1 (9)			
Approach of thoracotomy, *n* (%)													
Right anterolateral thoracotomy	13 (23)		8 (62)		5 (38)				6 (46)	7 (54)			
Left anterolateral thoracotomy	32 (57)		7 (22)		25 (78)				30 (94)	2 (6)			
Right and left anterolateral thoracotomy	3 (5)		0 (0)		3 (100)				3 (100)	0 (0)			
Clamshell thoracotomy	5 (9)		1 (20)		4 (80)				5 (100)	0 (0)			
Sternotomy	2 (4)		1 (50)		1 (50)				1 (50)	1 (50)			
Subxiphoid drainage	1 (2)		1 (100)		0 (0)				0 (0)	1 (100)			
Concomitant laparotomy, n (%)	24 (43)		5 (21)		19 (79)		0.153		21 (88)	3 (18)			0.319
Location thoracotomy, *n* (%)													
Pre-hospital	2 (4)		0 (0)		2 (100)		**0.018**		2 (100)	0 (0)			**0.003**
Emergency room	22 (39)		3 (14)		19 (86)				22 (100)	0 (0)			
Operating room	32 (57)		15 (47)		17 (53)				21 (66)	11 (34)			

*P*-values in bold denote significant *p*-values

*GCS*  Glasgow coma scale, *IQR*  interquartile range, *ISS* injury severity score, *PEA*  pulseless electrical activity; *SD* standard deviation, *SOL* sign of life

^a^3 missing

^b^ 4 missing

^c^5 missing

### Patient conditions upon arrival in the ED

**Fig. 2:**
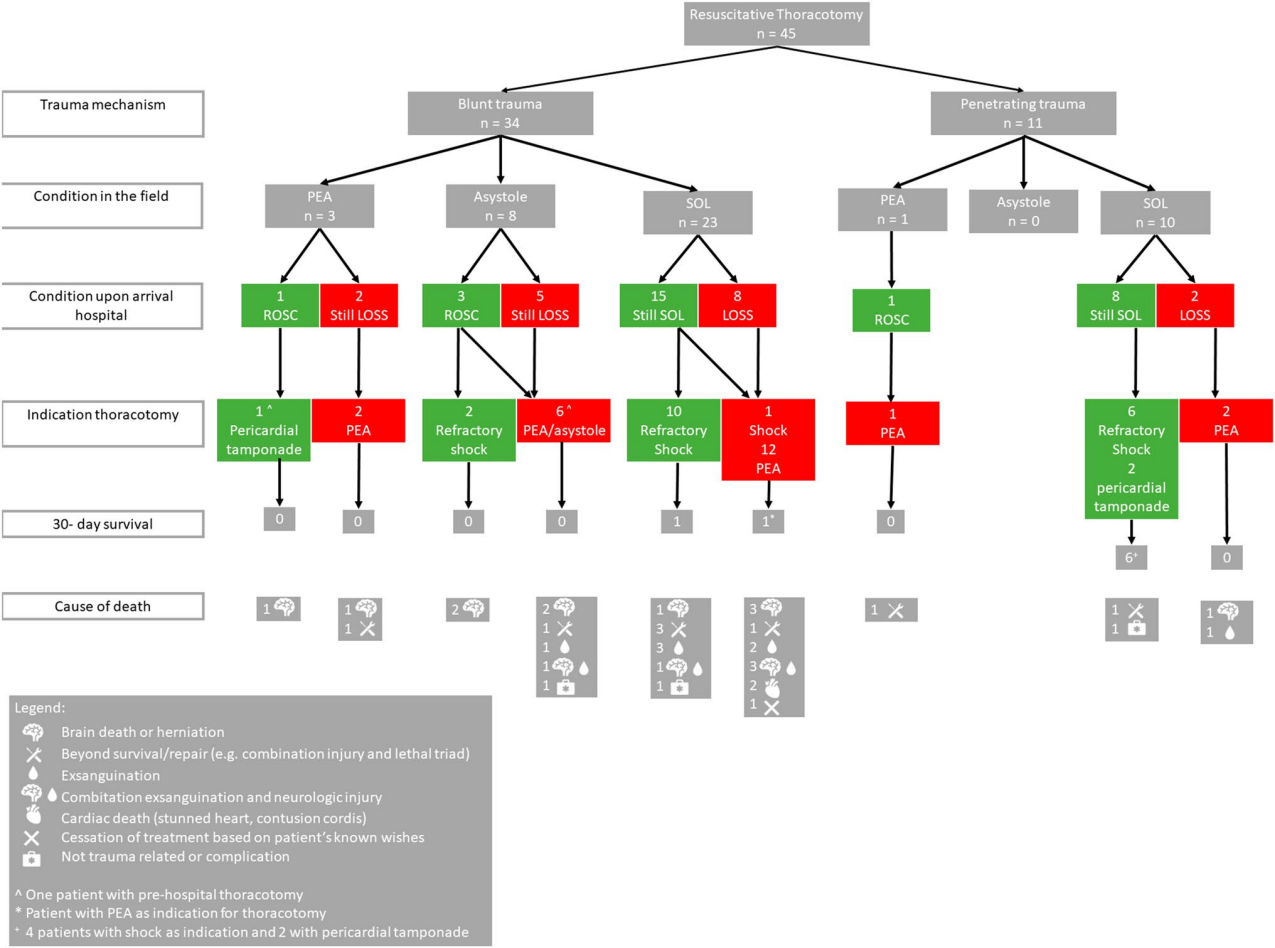
Flowchart of patients who underwent a resuscitative thoracotomy, including the indication for the thoracotomy and the outcomes

**Fig. 3:**
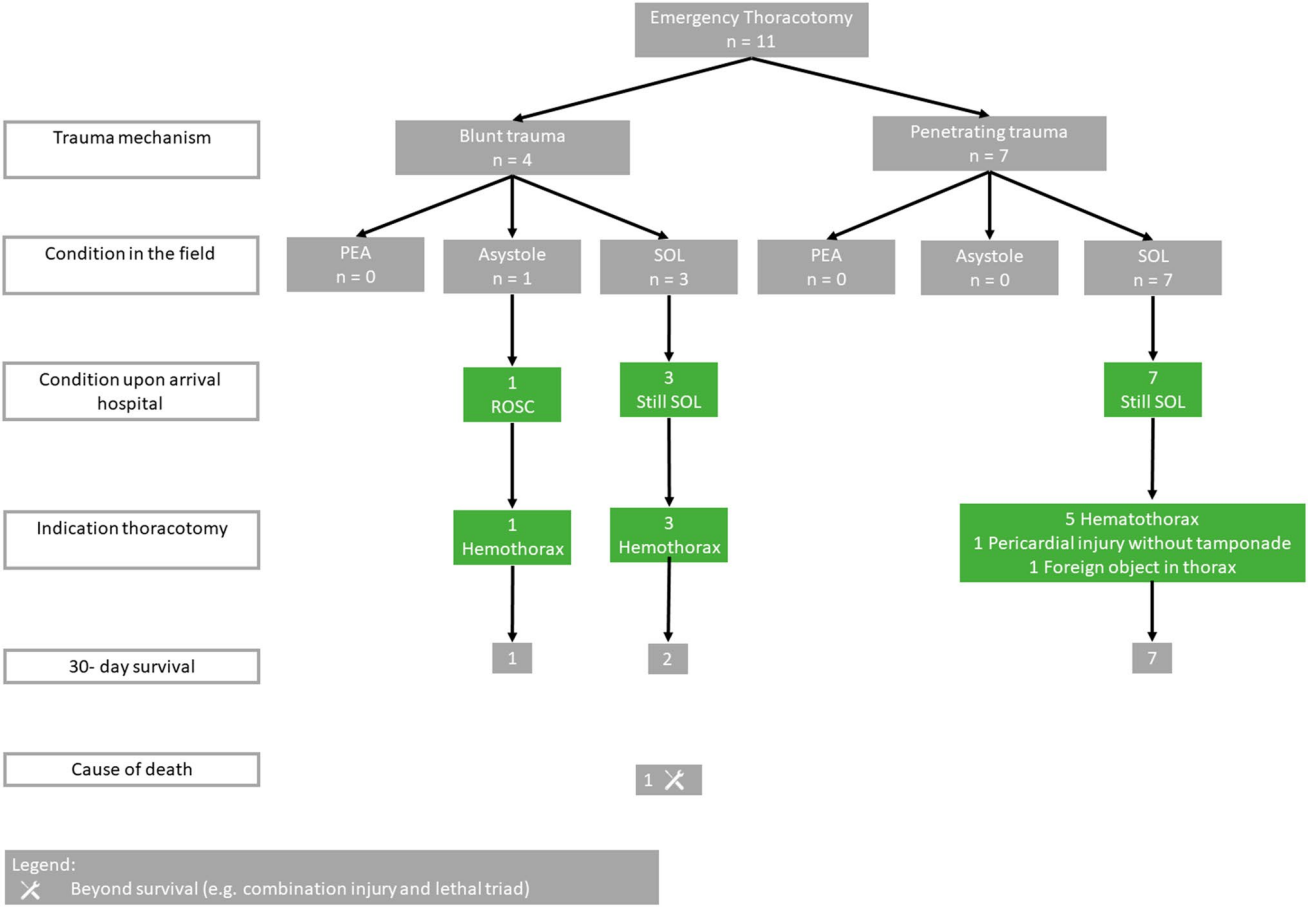
Flowchart of patients who underwent an emergency thoracotomy, including the indication for the thoracotomy and the outcomes

A total of 39 patients (70%) presented with (any) SOL at the hospital, eight patients (14%) had PEA and nine patients (16%) an asystole. A total of ten patients (18%) received CPR in the pre-hospital phase, of which three patients had return of spontaneous circulation (ROSC) upon arrival at the hospital, two patients had persisting PEA and five patients persisted in asystole (Fig. 2 and Fig. 3). The other five patients with PEA and two patients with asystole at presentation in the ER did not receive CPR in the pre-hospital phase. Vitals upon arrival can be found in Table 1 and laboratory results upon in Appendix 1 (ESM).

### Thoracotomy

The most common indication for a resuscitative thoracotomy was refractory shock due to PEA or asystole (*n* = 23, 51%), followed by severe (hemorrhagic) shock (*n* = 19, 42%) (Fig. 2). An emergency thoracotomy in hemodynamic (transient) responding patients was most often performed to treat a massive hemothorax with persistent blood loss (*n* = 9, 82%) (Table 1 and Fig. 3). Resuscitative thoracotomies were significantly more often performed after blunt trauma compared to emergency thoracotomies (89% vs. 11%; *p* = 0.013) (Table 1). One thoracotomy was performed by the helicopter emergency medical services in the pre-hospital setting, this patient had PEA after a stab wound to the thorax. The patient arrived with SOL to the hospital, but died the same day as result of the injuries combined with the lethal triad.

### Outcomes

Of the 56 patients, 18 patients (32%) survived the first 30 days after a resuscitative or emergency thoracotomy. Seven patients (39%) developed a complication, of which five infections, one re-bleed requiring an additional surgery and one patient, who also underwent a laparotomy, developed an ileus. Five patients underwent a secondary surgical procedure to treat their thoracic injuries. All 18 survivors had a Glasgow Outcome Scale of 5 out of 5. There were no differences, besides survival, in outcomes for patients who underwent a resuscitative or emergency thoracotomy (Table 2).

The cause of death of the 38 patients who died can be found in Fig. 2, Fig. 3 and Table 2. Two deaths (5%) were deemed as potentially preventable (Table 2), who died of exsanguination: one patient from a bleeding of the subclavian artery and one patients from bleeding of the hepatic and portal veins, which both might have been survivable if controlled earlier on. Although limited in numbers, every resuscitative thoracotomy in patients with blunt trauma and PEA or asystole in the pre-hospital phase was futile (0 survivors out of 11 patients), of those patients 7 (64%) had concomitant severe neuro-trauma (Fig. 2, 3).

**Table 2:** Differences in outcomes of patients undergoing resuscitative thoracotomy versus emergency thoracotomy

	Total *n* = 56	Resuscitative thoracotomy *n* = 45 (80%)	Emergency thoracotomy *n* = 11 (20%)	*p *value
Survived first 30 days, *n* (%)	18 (32)	8 (18)	10 (91)	** < 0.001**
Length of hospital stay in days, median (IQR)	11 (8–27)	16 (7–28)	11 (10–12)	0.824
ICU admission, *n* (%)	13 (72)	7 (88)	6 (60)	0.314
Length of ICU stay in days, median (IQR)	2 (1–8)	2 (2–8)	1 (1–15)	0.416
Complications, *n* (%)	7 (39)	5 (11)	2 (20)	0.145
Infection^a^	5 (72)	3 (60)	2 (100)	
Rebleeding^b^	1 (14)	1 (20)	0 (0)	
Ileus	1 (14)	1 (20)	0 (0)	
Second surgery for thorax trauma necessary, *n* (%)	5 (28)	2 (25)	3 (30)	1.000
Time to second surgery in days, mean ± SD	1 (1–2)	1	2 (1–3)	
Deceased within first 30 days (%)	38 (68)	37 (82)	1 (9)	** < 0.001**
Location where patient died, *n* (%)				
Emergency department	12 (32)	12 (32)	0 (0)	
Operating Room	11 (29)	10 (27)	1 (100)	
Intensive care unit	15 (39)	15 (40)	0 (0)	
Survived the first day after trauma, *n* (%)	8 (21)	8 (22)	0 (0)	
Cause of death and assumed survivability, *n* (%)				
Non-survivable	24 (63)	24 (65)	0 (0)	
*Brain death or herniation*	*11 (46)*	*11 (46)*	0 (0)	
*Uncontrollable bleeding from multiple sources*	*5 (21)*	*5 (21)*	*0 (0)*	
*Combination of 1 and 2*	*5 (21)*	*5 (21)*	*0 (0)*	
*Cardiac cause (e.g., stunned heart, contusion cordis with asystole)*	*3 (12)*	*3 (12)*	*0 (0)*	
Beyond survival (= injuries survivable as individual injury, however non-survivable due to accompanying factors)	8 (21)	7 (19)	1 (100)	
Cessation of treatment based on patients known wishes	1 (3)	1 (3)	0 (0)	
Possible preventable death	2 (5)	2 (5)	0 (0)	
Death not directly trauma-related^c^	2 (5)	2 (5)	0 (0)	
Complication (myocardial infarction 4 days after trauma)	1 (3)	1 (3)	0 (0)	

*P*-values in bold denote significant *p*-values

*ICU*  Intensive Care Unit, *SD*  Standard Deviation

^a^Peritonitis (*n* = 1), wound infection (*n* = 1), Back abscess (*n* = 1), mediastinitis (*n* = 1), pneumonia (*n* = 1)

^b^bleeding from subcutaneous artery.

^c^Decompensated liver cirrhosis after thorax trauma (*n* = 1), trauma was caused by myocarditis with myocardial infarction, which also was cause of death (*n* = 1)

### Factors associated with 30-day survival

Factors associated with higher 30-day survival rates were penetrating trauma compared to blunt trauma (*p* < 0.001), SOL upon presentation to the hospital compared to PEA or asystole (*p* = 0.005), a maximum GCS score of 15 (*p* < 0.001) and a thoracotomy performed in the OR compared to a thoracotomy in the field or in ED (*p* = 0.018) (Table 1).

## Discussion

This study found an overall 30-day survival rate of 32% for trauma patients who underwent either a resuscitative or emergency thoracotomy. Factors associated with 30-day survival in this study were: penetrating trauma, SOL upon presentation, higher GCS upon presentation and a thoracotomy performed in the OR compared to the ED or within the field. The latter two factors indicate that the opportunity of resuscitation prior to the thoracotomy, enabling enough time for safe transport to the operating room, increases survival. Survivors all had a maximum neurological recovery. All thoracotomies performed in patients after blunt trauma with PEA or asystole *before* arrival at the ED were futile.

Within this study, there was a substantial difference between the mortality rate of the resuscitative and emergency thoracotomy. Within current literature, the terms resuscitative and emergency thoracotomy are often interchangeable used without clear definition. For example, Panossian et al. reported on so-called ‘emergency resuscitative’ thoracotomies, but excluded explorative thoracotomies for hemorrhage control and Segalini et al. described emergency thoracotomies but includes thoracotomies both aimed at patients with traumatic cardiac arrest as patients with massive hemothorax [18, 19]. In this study, the decision was made to differentiate between both terms, especially since it was hypothesized that the outcomes would significantly differ. Studies limited to resuscitative thoracotomies reported mortality rates ranging from 5 to 19%, with rates as low as 1% in case of patients arriving without any SOL, while studies including both resuscitative as emergency thoracotomies report mortality rates ranging from 8 up to 33% [10, 14, 18–21]. In the present study, clear definitions of resuscitative and emergency thoracotomies were used, based on the physiological derangement of the patient and their hemodynamic response to resuscitative measures. As a result, resuscitative thoracotomies were performed in patients in extremis, without or disappearing SOL. In contrast, emergency thoracotomies were performed in patients with life-threatening thoracic injuries but who sustained an adequate circulatory status (mostly with ongoing resuscitation). The latter is also represented in the location where the procedure was performed, as these patients were hemodynamically stable enough to be transferred to the OR.

One of the most important factors associated with survival in this study was trauma mechanism. Trauma mechanism is already a factor greatly influencing the decision to perform a resuscitative thoracotomy due to the known difference in survival rates between blunt and penetrating trauma. Multiple meta-analyses have been published to support this finding, describing mortality rates for penetrating trauma varying from 8 to 22% and for blunt trauma rates varying from 2 to 7% [3, 4, 11, 14]. Interestingly, a meta-analysis including only European studies showed substantial higher survival rates for both blunt and penetrating trauma (blunt: 25%, penetrating 62%) compared to previous literature [8]. Moreover, the current study also showed greater overall survival rates in penetrating trauma (72%) compared to blunt trauma (23%) with rates similar to the European meta-analysis when both resuscitative and emergency thoracotomies were included. Possibly more patients presenting in extremis after penetrating trauma underwent a resuscitative thoracotomy, compared to patients after blunt trauma. It is tempting to speculate that the threshold for a resuscitative thoracotomy is higher in patients after blunt trauma in PEA or asystole upon arrival. This hypothesis is in concordance with the debate concerning the indication for thoracotomy in literature. In an article from Aseni et al. (2020), the indication is categorized in three categories: accepted, selected and rare indications. The accepted indications are penetrating injuries in the ‘cardiac box’ with profound refractory shock and poor hemodynamic parameters but with SOL. The selective indication is a patient with a penetrating thoracic injury arriving without SOL but received less than 15 min of CPR before return of SOL. The rare indication is the patient with blunt injuries arriving with SOL but with a witnessed loss of SOL in the ED requiring less than 10 min of CPR. Due to the low survival rate reported for blunt trauma, some trauma centers question the latter indication and are more reserved in performing a resuscitative thoracotomy in those latter patients [14]. The Eastern Association for the Surgery of Trauma (EAST) recommends a thoracotomy after blunt trauma only in case of patients presenting with PEA, but with pupillary response, and strictly recommends against the performance of a thoracotomy in patients presenting after blunt trauma without SOL [22]. This recommendation is in line with the study by Panossian et al., which studied a large nationwide database in the United States, and our study, since in both all patients with blunt trauma and without SOL before arrival did not survive [19]. In our study, most of those patients died because of brain death or herniation and not exsanguination.

The results of this study should be interpreted carefully because of its limitations. First, its retrospective nature has inevitably led to missing data (e.g., full medical history, CPR times, blood test results, FAST assessment records). This can largely be attributed to patients being presented in extremis in the ED, which makes complete data collection challenging. Blood test results, for example, are missing in 7–22 cases (depending on which blood test) which may have introduced selection bias despite the statistically significant outcomes. Furthermore, this retrospective study is underpowered, as are most of the studies in the available literature due to the relatively low occurrence of these severely injured patients. As a result, the conclusions that can be drawn from non-significant outcomes are limited.

## Conclusion

This study found a 30-day survival rate of 32% for resuscitative and emergency thoracotomies, all with good neurological recovery. Factors associated with survival were related to the trauma mechanism, the indication of the thoracotomy and response to resuscitation prior to the thoracotomy (for instance, if resuscitation enables enough time for safe transport to the operating room, survival chances increase). Resuscitative thoracotomies after blunt trauma in combination with loss of SOL before arrival at the ED were in all cases futile, in nearly all cases due to concomitant neuro-trauma. However, it could still be contemplated to perform a resuscitative thoracotomy as an extreme last resort depending on a patient’s age and/or comorbidities. In contrast, in patients presenting in extremis after penetrating trauma survival rate was 74%. In surviving patients, neurological and functional outcome was considered good.

## Supplementary Information

Below is the link to the electronic supplementary material.

Supplementary file1
