# Supplementary material for: Outcomes of the resuscitative and emergency thoracotomy at a Dutch level-one trauma center: are there predictive factors for survival?
Source: Eur J Trauma Emerg Surg. 2022 Jun 17;48(6):4877–87. doi: 10.1007/s00068-022-02021-x (PMC9712378; doi:10.1007/s00068-022-02021-x)
Supplement: Supplementary file 1 — Supplementary file1 [file 68_2022_2021_MOESM1_ESM.pdf]

Appendix 1 Laboratory results of patients who underwent an resuscitative or emergency thoracotomy

Laboratory results upon arrival hospital	Total n = 56 (100%)	Survived first 30 days n = 18 (32%)	Deceased within first 30-days n = 38 (68%)	Resuscitative thoracotomy n = 45 (80%)	Emergency thoracotomy n = 11 (20%)
Hemoglobin in mmol/L [a], median (IQR)	7.2 (5.5 – 8.1)	7.8 (6.0 – 8.6)	7.0 (5.1 – 7.6)	7.2 (5.1 – 8.0)	7.1 (6.1 – 8.6)
< 7.4 mmol/L, n (%)	*28 (56)*	*8 (29)*	*20 (71)*	*22 (79)*	*6 (21)*
Hematocrit in % [b], median (IQR)	34 (26 – 39)	37 (29 – 41)	34 (24 – 38)	34 (24 – 38)	36 (29 – 41)
<36%, n (%)	*28 (57)*	*7 (25)*	*21 (75)*	*23 (82)*	*5 (18)*
Leukocytes in x10^9/L[c], median (IQR)	12 (8 – 19)	15 (11 – 19)	10 (6 – 18)	12 (7 – 18)	15 (11 – 20)
>10 x10^9/L, n (%)	*23 (62)*	*13 (57)*	*10 (43)*	*16 (70)*	*7 (30)*
Thrombocytes in x10^9/L [d], median (IQR)	149 (113 – 237)	211 (154 – 277)	122 (82 – 159)	131 (112 – 211)	174 (119 – 269)
<150 x10^9/L, n (%)	*20 (50)*	*4 (20)*	*16 (80)*	*15 (75)*	*5 (25)*
PT in seconds [e], median (IQR)	22 (15 – 34)	16 (13 – 22)	27 (17 – 57)	22 (17 – 35)	16 (15 – 23)
>13 seconds, n (%)	*29 (85)*	*10 (34)*	*19 (66)*	*22 (76)*	*7 (24)*
aPTT in seconds [f], median (IQR)	53 (36 – 111)	33 (30 – 42)	72 (54 – 120)	60 (38 – 120)	36 (30 – 74)
>34 seconds, n (%)	*25 (76)*	*6 (24)*	*19 (76)*	*20 (80)*	*5 (20)*
pH[g], median (IQR)	7.06 (6.95 – 7.32)	7.33 (7.09 – 7.37)	7.00 (6.87 – 7.15)	7.03 (6.95 – 7.20)	7.33 (7.11 – 7.38)
<7.37, n (%)	*42 (89)*	*12 (29)*	30 (71*)*	*35 (83)*	*7 (17)*
Bicarbonate in mmol/L [g], median (IQR)	17 (12 – 22)	19 (16 – 22)	14 (11 – 21)	16 (11 – 21)	20 (16 – 23)
<22 mmol/L, n (%)	*35 (74)*	*10 (29)*	*25 (71)*	*29 (83)*	*6 (17)*
Base deficit in mmol/L [h], median (IQR)	11 (4 – 19)	8 (4 – 14)	15 (7 – 21)	14 (7 – 20)	6 (3 – 11)
>3 mmol/L , n (%)	*37 (80)*	*12 (32)*	*25 (68)*	*30 (81)*	*7 (19)*
>15 mmol/L, n (%)	*15 (33)*	*1 (7)*	*14 (93)*	*13 (87)*	*2 (13)*
Lactate in mmol/L [e], median (IQR)	7.7 (4.2 – 10.9)	6.1 (4.4 – 10.4)	9.3 (3.9 – 15.0)	9.3 (4.4 – 13)	5.2 (4.2 – 6.5)
>2.2 mmol/L, n (%)	*32 (94)*	*13 (41)*	*19 (59)*	*25 (78)*	*7 (22)*
pCO2 in mmHg [g], median (IQR)	49 (42 – 61)	42 (35 – 56)	56 (45 – 65)	52 (44 – 63)	43 (34 – 50)
>45 mmHg, n (%)	*30 (64)*	*7 (41)*	*23 (74)*	*25 (83)*	*5 (17)*
Potassium in mmol/L[b], median (IQR)	4.1 (3.6 – 5.3)	3.6 (3.5 – 4.0)	4.9 (3.8 – 6.6)	4.4 (3.6 – 6.1)	3.6 (3.5 – 3.9)
>5.0 mmol/L, n (%)	*15 (31)*	*0 (0)*	*15 (100)*	*15 (100)*	*0 (0)*

Abbreviations: aPPT = Activated Partial Thromboplastin Time; IQR = Interquartile Range; PT = Prothrombin Time.
Missing variables: [a] 6 missing; [b] 7 missing; [c] 19 missing; [d] 16 missing; [e] 22 missing; [f] 23 missing; [g] 9 missing; [h] 10 missing.
*References values: Hemoglobin 7.4 – 9.6 mmol/L, Hematocrit 36 – 46%, Leukocytes 4.0 – 10.0 x10^9/L, Thrombocytes 150 – 450 x 10^9/L, PT 10-13 seconds, aPTT 24 – 34 seconds, pH 7.37 – 7.45, Bicarbonate 22 – 29 mmol/L, Base deficit -3.0 – 3.0 mmol/L, Lactate 0.0-2.2 mmol/L, pCO2 35 – 45 mmHg, Potassium 3.8 – 5.0 mmol/L.
